# Local adaptation with gene flow in a highly dispersive shark

**DOI:** 10.1111/eva.13628

**Published:** 2023-12-20

**Authors:** Juliana D. Klein, Simo N. Maduna, Matthew L. Dicken, Charlene da Silva, Michelle Soekoe, Meaghen E. McCord, Warren M. Potts, Snorre B. Hagen, Aletta E. Bester‐van der Merwe

**Affiliations:** ^1^ Molecular Breeding and Biodiversity Research Group, Department of Genetics Stellenbosch University Stellenbosch South Africa; ^2^ Department of Ecosystems in the Barents Region, Svanhovd Research Station Norwegian Institute of Bioeconomy Research—NIBIO Svanvik Norway; ^3^ KwaZulu‐Natal Sharks Board Umhlanga Rocks South Africa; ^4^ Institute for Coastal and Marine Research (CMR), Ocean Sciences Campus Nelson Mandela University Gqeberha South Africa; ^5^ Department of Forestry, Fisheries and Environment Rogge Bay South Africa; ^6^ Division of Marine Science Reel Science Coalition Cape Town South Africa; ^7^ South African Shark Conservancy Hermanus South Africa; ^8^ Canadian Parks and Wilderness Society Vancouver British Columbia Canada; ^9^ Department of Ichthyology and Fisheries Science Rhodes University Makhanda South Africa; ^10^ South African Institute for Aquatic Biodiversity Makhanda South Africa

**Keywords:** adaptive divergence, elasmobranchs, genotype–environment association, population genomics, seascape genomics

## Abstract

Adaptive divergence in response to environmental clines are expected to be common in species occupying heterogeneous environments. Despite numerous advances in techniques appropriate for non‐model species, gene–environment association studies in elasmobranchs are still scarce. The bronze whaler or copper shark (*Carcharhinus brachyurus*) is a large coastal shark with a wide distribution and one of the most exploited elasmobranchs in southern Africa. Here, we assessed the distribution of neutral and adaptive genomic diversity in *C. brachyurus* across a highly heterogeneous environment in southern Africa based on genome‐wide SNPs obtained through a restriction site‐associated DNA method (3RAD). A combination of differentiation‐based genome‐scan (outflank) and genotype–environment analyses (redundancy analysis, latent factor mixed models) identified a total of 234 differentiation‐based outlier and candidate SNPs associated with bioclimatic variables. Analysis of 26,299 putatively neutral SNPs revealed moderate and evenly distributed levels of genomic diversity across sites from the east coast of South Africa to Angola. Multivariate and clustering analyses demonstrated a high degree of gene flow with no significant population structuring among or within ocean basins. In contrast, the putatively adaptive SNPs demonstrated the presence of two clusters and deep divergence between Angola and all other individuals from Namibia and South Africa. These results provide evidence for adaptive divergence in response to a heterogeneous seascape in a large, mobile shark despite high levels of gene flow. These results are expected to inform management strategies and policy at the national and regional level for conservation of *C. brachyurus* populations.

## 1 INTRODUCTION

Local adaptation to environmental conditions is one of the main drivers of evolution with organisms adapting to biotic and abiotic factors that are changing over time and space (Kawecki & Ebert, 2004). In species that occupy heterogeneous environments this process can lead to adaptive divergence of populations on different geographic scales even in the face of neutral homogeneity and an absence of physical barriers to gene flow (Attard et al., 2018; Gleason & Burton, 2016).

In non‐model species, the detection of outlier loci associated with adaptive divergence relies on advances in next‐generation‐sequencing methods that enable genotyping of single nucleotide polymorphisms (SNPs) across the genome such as RADseq or ddRAD‐seq (Andrews et al., 2016; Peterson et al., 2012). These developments now allow the integration of genomic and environmental data to directly associate allele frequencies of SNPs highly correlated with environmental variables (Manel et al., 2003; Selkoe et al., 2016). This landscape or seascape genomics framework can provide a better understanding of how dispersal, geography and environment interact to shape genetic variation and to delineate relevant ecological and evolutionary conservation units (Fraser & Bernatchez, 2001). Moreover, this framework enables one to address previously intractable research areas, such as assessing populations' genomic vulnerability and forecasting of adaptive capacity to reveal potential mismatches under a rapidly changing climate (Nielsen et al., 2021; Vranken et al., 2021).

**FIGURE 1:**
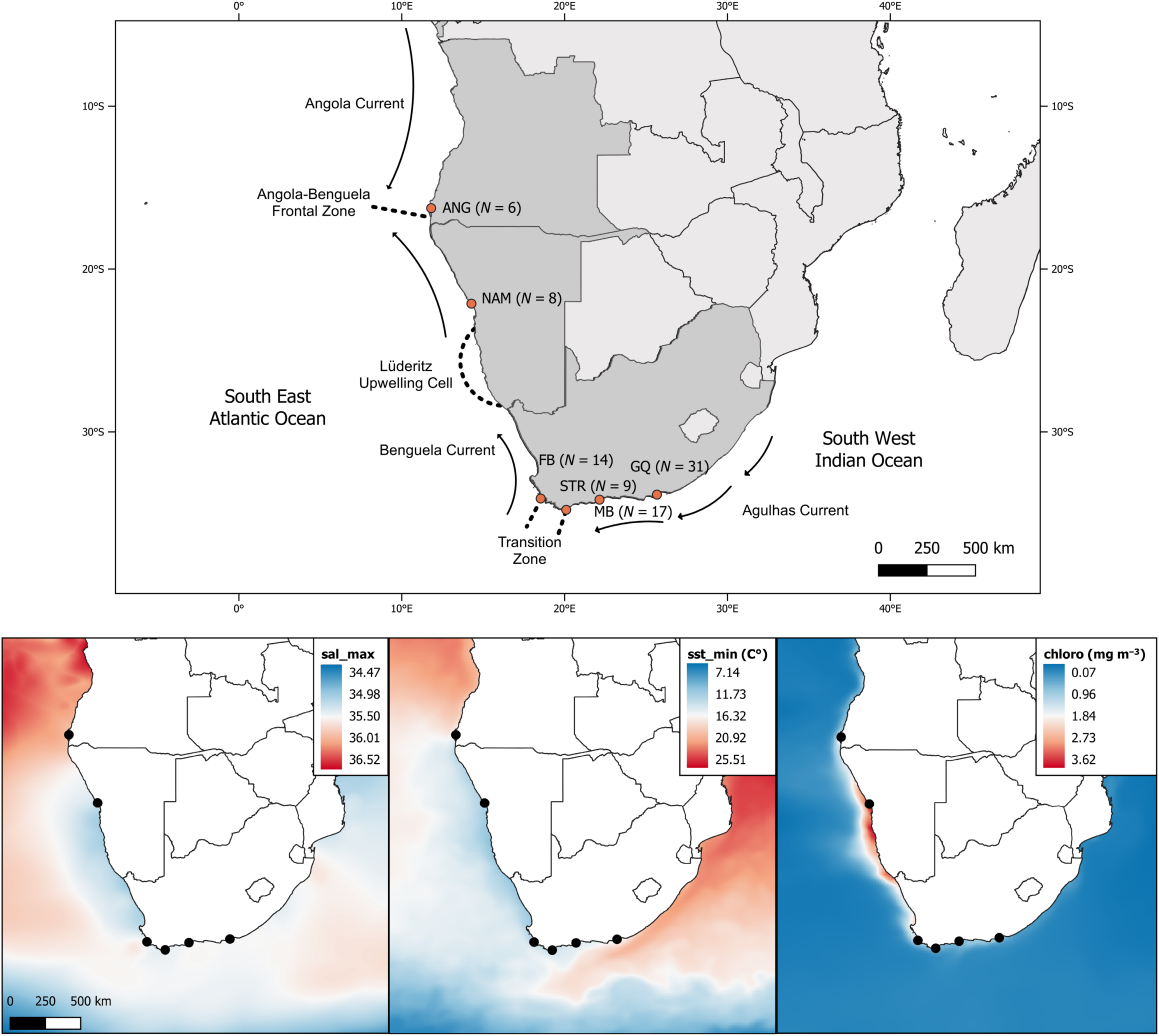
Top: Map of Southern Africa including main oceanographic features and sampling sites of bronze whaler sharks *Carcharhinus brachyurus* across South Africa, Namibia and Angola. ANG, Tombua, Angola; FB, False Bay; GQ, Gqeberha; MB, Mossel Bay; NAM, Henties Bay, Namibia; STR, Struisbaai. Bottom: Variability in environmental variables used in redundancy analysis (maximum salinity, minimum sea‐surface temperature, chlorophyll concentration) along the Southern African coast. Sampling sites are indicated as black dots.

The southern African coastline provides a natural laboratory to study local adaptation due to its highly dynamic environment, where the Agulhas and Benguela currents and their convergence shape a diversity of biogeographic regions. The warm Agulhas Current creates a subtropical and warm‐temperate zone along the East and South Coast of South Africa whereas the West Coast and Namibia are influenced by the Benguela current and wind‐driven upwelling systems, creating a cool‐temperate, highly productive coastal area (Briggs & Bowen, 2012; Lutjeharms & Meeuwis, 1987; Nelson & Hutchings, 1983). Off Angola, the warm Angola Current creates a tropical and sub‐tropical coastal zone and a perennial, oceanographic barrier where its southern boundary meets the Benguela Current (the Angola‐Benguela‐Frontal‐Zone (ABFZ), Figure 1) (Shannon et al., 1987). Species occupying the different biogeographic regions are exposed to different selective pressures, which can lead to adaptive differences in genetic variation among populations, even in the face of high gene flow (Nielsen et al., 2020; Schulze et al., 2020; Teske et al., 2019).

In comparison to terrestrial ecosystems, the seascape is typically characterized by a lack of clear barriers and in combination with high dispersal capabilities and large populations sizes in marine animals, higher gene flow and lower absolute levels of differentiation are often observed (Selkoe et al., 2016; Xuereb et al., 2018). However, complex interactions between spatial and environmental processes, mediated by a species' particular life‐history traits can drive adaptive divergence on different spatial and temporal scales when compared with neutral genetic variation. Genome‐wide associations with environmental variables have been shown for many marine taxa, including invertebrates (Sandoval‐Castillo et al., 2017; Xuereb et al., 2018), teleost fishes (Antoniou et al., 2023; Boulanger et al., 2021; Cayuela et al., 2020) and marine mammals (Barceló et al., 2022; Pratt et al., 2022). In these studies, several environmental variables have emerged as powerful drivers of adaptive divergence, including for instance sea‐surface temperature, oxygen and salinity. Although few studies have examined genome‐wide associations with environmental variables in elasmobranchs, Delaval et al. (2022) used an integrated population‐seascape genomics framework to identify candidate SNPs linked to environmental variables relevant under a changing climate for the habitat‐restricted blue skate, *Dipturus batis*. Expanding this knowledge is essential for improving the biodiversity monitoring and management of elasmobranchs as they are one of the most threatened vertebrate groups globally with an estimated one‐third of species being driven to extinction mainly through overfishing (Dulvy et al., 2021). High levels of overexploitation of elasmobranchs are a result of the demand for consumer products such as fins, gill plates and liver oil traded internationally, as well as increased demand for shark meat for consumption due to dwindling teleost fish stocks (Cardeñosa et al., 2022; Seidu et al., 2022; Tyabji et al., 2022).

The bronze whaler or copper shark *Carcharhinus brachyurus* is a large (up to 3 m total length) coastal shark with cosmopolitan but patchy distribution (Compagno et al., 2005). This species is considered highly valuable in the shark meat trade and is not only threatened by exploitation by a range of directed and non‐directed fisheries throughout its range but also by coastal development due to its affinity to coastal areas (da Silva et al., 2015; Drew et al., 2019; Lucifora et al., 2005; Rogers et al., 2013). The life history parameters of this species vary across its distribution but like most elasmobranchs, the species is characterized by low fecundity (mean litter size = 15, range 8–20) (Cliff & Dudley, 1992) and late maturity (200–224 cm PCL for males, 215–270 cm PCL for females) (Drew et al., 2016; Lucifora et al., 2005; Smale, 1991). *C. brachyurus* is currently listed as Vulnerable by the IUCN (Huveneers et al., 2020) with a globally decreasing population size trend. In contrast, the local stock status in South Africa appears to be improving with recommendations of uplisting the species status to Least Concern (DFFE, 2022), indicating that southern Africa may represent one of the remaining hotspots for this species. In the region, the species is distributed from Angola in the South‐East Atlantic to the South‐West Indian Ocean off the East Coast of South Africa (Compagno et al., 2005).

To date, the population structure of *C. brachyurus* in southern Africa remains unclear. Early hypotheses by Walter and Ebert (1991) suggested that two distinct populations exist along the southern African coast, one from the Western Cape eastwards and the other off Namibia north of Walvis Bay. This was based on the species distribution disjunction previously observed in the Lüderitz area and an apparent difference in breeding seasonality. The Lüderitz area's influence on genetic breaks and species divergence due to the cold‐water upwelling formation characterizing the region (Lutjeharms & Meeuwis, 1987) is known for other elasmobranchs and marine organisms (Henriques et al., 2014, 2015; Hull et al., 2019). However, given the highly migratory behaviour of this species and based on previous investigations, extensive gene flow along coastal areas is expected (Benavides et al., 2011; Huveneers et al., 2021; Junge et al., 2019). High levels of gene flow may counteract the effects of selective pressures by homogenizing allele frequencies and “swamping” of adaptive alleles (Lenormand, 2002), but it is unknown how these forces interact in this species. However, there is now mounting evidence from diverse terrestrial (Clark et al., 2022; Hämälä & Savolainen, 2019; Muir et al., 2014) and aquatic taxa (Cayuela et al., 2020; Fitzpatrick et al., 2015; Salloum et al., 2022) that local adaptation is not necessarily impeded by gene flow. Patterns suggestive of local adaptation have also been observed in marine fish that occupy the highly heterogenous environment of the southern African coast (Henriques et al., 2016; Schulze et al., 2020).

An assessment of genome‐wide and adaptive variation on a local and regional scale is therefore needed for *C. brachyurus* to better understand the degree of genetic connectivity in this species. Here, we employed the 3RAD approach (Bayona‐Vásquez et al., 2019), an extension of the original double‐digest RADseq protocol (Peterson et al., 2012) to comprehensively assess the regional population genomics of *C. brachyurus* across a heterogeneous environment, which includes the South‐East Atlantic and South‐West Indian Ocean. In particular, we aimed to address two specific hypotheses. Firstly, we investigated the distribution of neutral genomic diversity to evaluate the degree of genetic connectivity of populations across the distribution of *C. brachyurus* in southern Africa. Based on the high dispersal abilities of *C. brachyurus* and the apparent lack of regional population structure in previous studies conducted elsewhere (Benavides et al., 2011; Junge et al., 2019), we hypothesized that gene flow along the sampled area is high with low levels of population differentiation. Given the high environmental heterogeneity across the study area, we further aimed to investigate patterns of local adaptation based on putatively adaptive SNPs. To achieve this, we used a combination of differentiation‐based and genotype–environment association (GEA) analyses to identify potential candidate SNPs. Levels of diversity and differentiation were then assessed separately for putatively neutral and adaptive SNP data. These findings are expected to be used to inform conservation, management strategies and policy at the national and regional level.

## 2 MATERIALS AND METHODS

### 2.1 Sampling, library preparation and SNP genotyping

Our study area encompasses the near‐complete distribution of *C. brachyurus* across southern Africa. We obtained 85 fin clips collected by recreational rock‐ and shore fishermen from 2014 to 2020 at six sampling sites distributed across three countries. These include (from southeast to northwest) South Africa (*N*_*site*_ = 4; Gqeberha (*N* = 31), Mossel Bay (*N* = 17), Struisbaai (*N* = 9) and False Bay (*N* = 14)), Angola (*N*_*site*_ = 1; Tombua (*N* = 6)) and Namibia (*N*_*site*_ = 1; Henties Bay (*N* = 8)) (Figure 1). Permits to collect fin clips for research purposes were granted by the Department of Forestry, Fishery and Environment (DFFE) in South Africa and from the Instituto Nacional de Investigação Pesqueira (INIP) in Angola. Ethical clearance was provided by the Research Ethics (Animal Care and Use) committee in the form of an Animal Notification with reference number #ACU‐2021‐21616.

Fin clips were preserved in 95% ethanol at room temperature until total genomic DNA was extracted using a standard cetyltrimethylammonium bromide (CTAB) protocol (Doyle & Doyle, 1987). Integrity of gDNA was assessed on a 0.8% agarose gel and potential protein or salt contamination were evaluated with a NanoDrop ND 2000 spectrophotometer (Thermo Fisher Scientific). A Qubit assay was used to determine DNA concentration and all samples were standardized to a concentration of 20 ng/μl. RADseq libraries were prepared using the Adapterama III library preparation protocol (Bayona‐Vásquez et al., 2019; their File S1), which is a modified version of double‐digest (dd)RAD (Peterson et al., 2012) that uses three enzymes for digesting genomic DNA (3RAD). We selected the 3RAD Design 2 which usese *MspI* (C|CGG) as the frequent cutter, *BamHI‐HF* (G|GATCC) as the rare cutter, and *ClaI* (AT|CGAT) as the third enzyme for suppressing phosphorylated ends in *MspI* recognition sites and inhibiting the formation of adapter dimers and DNA chimeras. As a reference or control in our laboratory setup for 3RADseq, we also tested the 3RAD Design 1 enzyme combination (*XbaI*, *EcoRI‐HF*, and *NheI*) from Bayona‐Vásquez et al. (2019). A total of 85 *C. brachyurus* samples were prepared with 299 samples from other projects. A detailed description of the laboratory procedures can be found in the File S1. A total of 576 samples (384 samples for D2 plus 192 samples for D1) were sequenced on two lanes of the Illumina HiSeq 4000 platform (i.e. 288 samples/lane). Because this did not achieve the desired coverage, the D2 libraries (384 samples) were re‐pooled and sent to the Oslo Hospital Node of the NSC for QC, size selection and re‐sequencing on one flowcell of the Illumina NovaSeq S4. Quality control of the raw data was conducted with fastqc v0.11.5 (https://www.bioinformatics.babraham.ac.uk/projects/fastqc/). To remove adapter sequences, reads were processed with cutadapt v1.9.1 (Martin, 2011) (F: AGATCGGAAGAGCACACGTCTGAACTCCAGTCAC, R: AGATCGGAAGAGCGTCGTGTAGGGAAAGAGTGT). Trimmed reads from both sequencing runs were merged before demultiplexing using *process_radtags* (with parameters ‐c ‐q ‐r ‐t 110 ‐w 0.15 ‐s 10) in stacks v2.59 (Catchen et al., 2011). At this stage, two samples were removed from the dataset due to a low number of reads retained. Loci were assembled de novo using the *denovo_map.pl* pipeline that runs each component of stacks. To determine suitable parameter values, a subset of samples (*N* = 14) was used to run the core pipeline several times, iterating over increasing values of the assembly parameters. Optimal parameters were then identified based on the *r80* rule (Paris et al., 2017; Rochette & Catchen, 2017). The final assembly was performed with a minimum of three reads required to form a primary stack (m = 3), two mismatches allowed between putative alleles in a locus (M = 2) and two mismatches between loci in the catalog (n = 2) (Table S1). After evaluation of the assembly, another sample was removed for which only a very small number of genotypes was called (<15,000). To limit the amount of missing data and ensure that loci are broadly shared across samples, the *populations* module was invoked again to retain only loci that were present in at least 60% of the samples (‐R 0.6) and to remove loci with maximum heterozygosity >0.5. The resulting SNP genotype dataset was then subjected to further filtering criteria in vcftools v0.1.14 (Danecek et al., 2011). Given our aim to investigate GEA and signatures of selection in a non‐model species, we chose more relaxed filtering criteria to retain an overall larger set of samples and SNPs and increase the power of GEA to detect loci under weak selection: SNPs with a minimum GQ of <20, minimum read depth <5 and minor allele count <3 were removed. Lastly, loci with genotype call rate <85% and individual samples with >40% missing data were removed and only one random SNP on each locus was kept. We also explored how different filtering strategies, in particular regarding minimum read depth, missing data and inclusion of rare alleles would affect our inferences regarding the extent of population structure and adaptive divergence. All results remained consistent regardless of specific filtering thresholds and criteria tested (data not shown), validating that results are not driven by a particular filtering strategy. The final dataset consisted of 81 samples genotyped across 26,299 SNPs with an average amount of missing data of 3.62%.

### 2.2 Identifying neutral and adaptive loci

We employed three different methods to detect outliers and candidate SNPs associated with environmental variability. First, a differentiation‐based outlier method was selected that identifies candidate SNPs based on above‐average differentiation. The R package outflank v0.2 (Whitlock & Lotterhos, 2015) was used which detects outliers under divergent selection by initially inferring the *F*_ST_ distribution from multiple loci and then fitting a *χ* ^2^ model to the centre of the distribution. The resulting null distribution is then used to detect outlier loci. Loci in the upper and lower 5% of the empirical distribution were trimmed as suggested by Whitlock and Lotterhos (2015) and large *F*_ST_ outliers in the upper tail were identified based on a False Discovery Rate (FDR) of 5% (qthreshold = 0.05). Preliminary analyses indicated no discernible population structure, hence the analysis was run with *K* = 1 and *K* = 6 (total number of sampling sites) to account for uncertainties regarding the number of clusters.

As a primary GEA approach, we chose to use Redundancy analysis (RDA). This method is used to identify associations between a specific allele, or locus genotype, and a set of environmental variables or factors hypothesized to drive selection. RDA is a powerful constrained ordination method, combining multiple linear regression and PCA, with less false‐positive detections than other methods over a range of selection levels, demographic scenarios, sampling designs and sample sizes (Capblancq et al., 2018; Forester et al., 2018). We first evaluated the role of spatial structure in shaping genomic variation by applying a spatial eigenfunction approach based on distance‐based Moran's Eigenvector Map (dbMEMs). These represent uncorrelated spatial variables that can be used in ordination analyses as explanatory variables (Borcard & Legendre, 2002). Location coordinates were used to compute marine geographical distances with the function *marineDistances* (https://github.com/jorgeassis/marineDistances), which uses a shortest‐path algorithm considering infinite resistance of landmasses. Marine distances were converted to a Euclidean distance matrix between all sampling sites. dbMEMs were then calculated with the package *adespatial* v0.3.20 with the default truncation threshold (Dray et al., 2023).

A total of 11 bioclimatic variables were considered, which included four measures of sea‐surface temperature (minimum, maximum, mean and range between 2002 and 2010) as well as four measures of salinity (minimum, maximum, mean and range between 1955 and 2006) at a resolution of ~1 km obtained from the marspec database (Sbrocco & Barber, 2013). Mean surface dissolved oxygen, pH and chlorophyll concentration for the period of 2000–2014 were retrieved from bio‐oracle v2.2 (Assis et al., 2018; Tyberghein et al., 2012) at a resolution of ~9.2 km. Site‐specific data were downloaded with the package sdmpredictors v0.2.14 (Bosch et al., 2017) (see mean values for sampled sites in Table S2). The genomic data was prepared by imputing missing data with the most common genotype at that locus. We then ran a global redundancy model including all spatial and bioclimatic variables and performed a stepwise (bidirectional) selection procedure with the function *ordistep* of the package *vegan* v2.6.4 (Oksanen et al., 2022) to identify the most important explanatory variables. None of the dbMEMs were selected as significant predictors and three bioclimatic variables, namely maximum salinity, minimum sea‐surface temperature and chlorophyll concentration were retained for the final model. None of the selected variables were highly correlated (Figure S1) and the variance inflation factor (VIF) for these predictors ranged from 1.26 to 1.59, hence no residual collinearity was assumed. Significance of the overall model and each axis was assessed through 1000 analysis of variance (ANOVA) permutations. Loci that showed loading ±3 standard deviation from the mean loading of significant RDA axes were regarded as candidates being under selection. The correlation of allele frequency of these candidate SNPs with each of the retained environmental variables was calculated to establish which variable each candidate SNP was most associated with.

As an alternative GEA approach, we employed a latent factor mixed‐effect model (LFMM) in the R package lea v3.8.0 (Frichot & François, 2015). LFMMS have a low rate of false positives, and perform well under a range of demographic scenarios and sampling regimes as well as complex population structure (Caye et al., 2019; Frichot et al., 2013). Although this approach can account for population structure using latent factors, none were included due to the lack of discernible structure observed for *C. brachyurus* in the current study (see Results). Hence, this approach is equivalent to a univariate linear regression. For this approach, we chose to reduce collinearity among environmental variables by summarizing the 11 variables through a PCA. To determine the number of PCs to retain, we applied the “broken stick criterion”, retaining only PCs with eigenvalues exceeding the value generated by a random distribution (King & Jackson, 1999). Here, only the first three PCs were retained for further analysis (Figure S2). For optimal computational speed, we used the newer *lfmm2* implementation that uses a least‐squares estimation method to compute LFMMs (Caye et al., 2019). After computing the model, we calculated the genomic inflation factor (GIF) and applied FDR control method by converting *p*‐values to *q*‐values with the package qvalue v.2.28.0 (Storey et al., 2022). SNPs with a FDR of <1% were considered as outliers.

All unique candidate and outlier SNPs detected by the three methods were considered our putatively “adaptive” SNPs whereas the rest of the SNPs were considered our “neutral” data. The following diversity and differentiation analyses were conducted on both datasets to separate neutral mutation‐drift processes from adaptive divergence.

### 2.3 Genetic diversity and population structure

Diversity indices including expected heterozygosity (*H*_E_), observed heterozygosity (*H*_O_), rarefactioned allelic richness (*A*_R_), and inbreeding coefficient *F*_IS_ were estimated with the R package diversity v1.9.90 (Keenan et al., 2013). To investigate population differentiation, we employed various methods: first we calculated population pairwise *F*_ST_ using the package hierfstat v0.5.11 (Goudet, 2005) with 95% confidence intervals generated under 1000 bootstrap intervals. The hypothesis of inter‐oceanic structure was tested using an AMOVA using the *ade4* method implemented in the package poppr v2.9.4 (Kamvar et al., 2014). For this analysis, sampling sites were pooled into their respective ocean groups (South‐West Indian Ocean (SWIO): GQ, MB, STR; South‐East Atlantic Ocean (SEAO): FB, NAM, ANG) to test how genetic diversity is partitioned into the different hierarchical levels. Significance was determined through 1000 permutations.

Next, we computed a Discriminant Analysis of Principal Components (DAPC; Jombart et al., 2010) in the package adegenet v2.1.10 (Jombart, 2008). For DAPCs without spatial prior information, we inferred the number of discrete genetic clusters using the *find*.*clusters()* function, which runs successive *K*‐means clustering with an increasing number of clusters (*K*). We ran 8 independent runs of the *find.clusters* function to identify sharp changes in the fit of models with different number of clusters based on the Bayesian information criterion (BIC) score. Because the search for an optimal number of clusters indicated that *K* = 1 (Figure S3a) for the neutral dataset, DAPC was computed on this data in a second run on clusters pre‐defined by sampling area at a spatial level. For all DAPC analyses, we determined the number of principal components to retain using the cross‐validation approach implemented by the function *xvalDapc()* with 100 repetitions.

Lastly, we examined population structure by calculating ancestry coefficients using a program based on sparse non‐negative matrix factorization (sNMF). This method estimates ancestry coefficients comparable to those obtained by other widely used programs such as admixture and structure, but is computationally faster and robust to departures from model assumptions such as Hardy–Weinberg equilibrium (Frichot et al., 2014). Individual admixture coefficients were estimated for 1–8 ancestral populations *K* with 10 independent replicates for each *K*. The cross‐entropy criterion was then used to determine the best *K* based on the prediction of masked genotypes. The sNMF analysis was implemented through the R package lea and resulting ancestry coefficients were visualized in barplots with the package pophelper v2.3.1 (Francis, 2017).

## 3 RESULTS

### 3.1 Characterization of 3RAD data

A total of 1,210,575,860 reads were generated during the two sequencing runs. After demultiplexing and initial filtering, 815,100,629 reads were retained, which were used to assemble loci using the *denovo_mapl.pl* pipeline in stacks. With the optimized parameters, the final assembly generated a total of 556,981 loci carrying 373,311 SNPs. After filtering and keeping only one SNP per locus, the final dataset consisted of 26,299 SNPs genotyped in 81 individuals.

### 3.2 Identification of candidate SNPs

**FIGURE 2:**
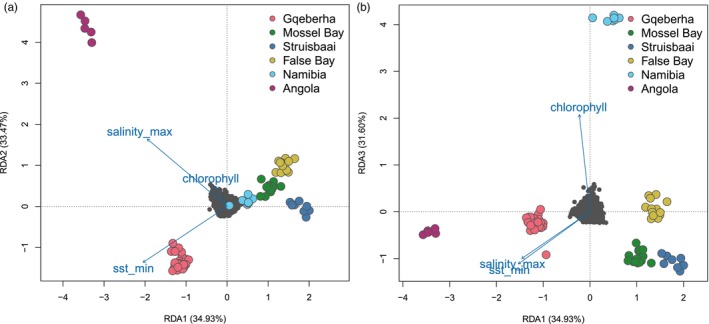
Redundancy analysis on 26,699 SNPs (grey filled circles in the centre) genotyped in 81 individuals with colors based on sampling site and three environmental predictors (minimum sea‐surface temperature, maximum salinity, mean chlorophyll concentration) depicted as blue vectors. Length of the vectors reflects the amount of variance in SNP genotypes explained by that variable and angles of vectors represent correlation between variables. Plot (a) shows constrained variation along axis 1 (34.93%) and axis 2 (33.47%), plot (b) shows constrained variation along axis 1 and axis 3 (31.60%).

The genome‐scan outflank detected the same putative outliers in both runs using *K* = 1 and *K* = 6. A total of 52 candidate SNPs were identified based on a *q*‐value threshold of 0.05 and no SNPs with atypically low *F*_ST_ values were detected. The full RDA model was significant (*p*‐value = 0.001, adjusted *R* ^2^ = 0.002) but testing each axis separately showed that only the first two axes were significant (ANOVA, *p*‐value = 0.001) which explained 68.40% of the constrained variation. Individuals sampled in Angola and Gqeberha showed strong positive correlations with maximum salinity and minimum sea‐surface temperature (Figure 2a,b). On axis 3, a positive correlation of genotypes from Namibia with mean chlorophyll concentration was observed (Figure 2b). For the identification of candidate SNPs we only considered loci on the first two significant constrained axes. A total of 201 unique outlier SNPs were detected across the two axes with 29 SNPs most strongly correlated with maximum salinity, 132 with minimum sea‐surface temperature, and 40 with mean chlorophyll concentration. For the LFMM analysis, the GIF of the first three PCs retained ranged from 1.07 to 1.40, indicating that the model was appropriately calibrated (Devlin & Roeder, 1999). Using a FDR <1%, 10 candidate SNPs were detected along axis 1, 28 along axis 2 and 12 SNPs along axis 3. which together explained 90.20% of the total variation.

There was considerable overlap among candidate SNPs detected by the different methods. Of the 304 candidate SNPs identified in total by all three methods, 29 SNPs were detected by RDA and LFMM, 23 by RDA and outflank and 18 were detected by outflank and LFMM. This resulted in a total of 234 unique SNPs detected by the three methods which where separated from the full dataset to create our adaptive dataset. The remaining 26,065 SNPs were considered as our neutral data.

### 3.3 Neutral and adaptive distribution of diversity and differentiation

Diversity estimates indicate low to moderate levels of genetic diversity with overall mean rarefactioned allelic richness of 1.535 and 1.416 for the neutral and adaptive data respectively (Table 1). Diversity estimates based on heterozygosity did not vary much among sampling sites for the neutral data with *H*_O_ and *H*_E_ ranging from 0.170 to 0.195 and from 0.149 to 0.167 respectively. Heterozygosity based on adaptive data showed more variability among sites with values ranging from 0.093 and 0.082 in Gqeberha to 0.476 and 0.330 in Angola and 0.082 and 0.330 for *H*_O_ and *H*_E_, respectively. Inbreeding coefficient *F*_IS_ and associated 95% CIs indicated significant outbreeding in all sampling sites for both neutral and adaptive data.

**TABLE 1:** Diversity indices estimated for each bronze whaler shark *Carcharhinus brachyurus* sampling site based on neutral and adaptive SNPs.

Neutral data							Adaptive data					
Sampling site	*A*_R_	*H*_O_	*H*_*E*_	*F*_IS_	*F*_IS_ low 95%	*F*_IS_ high 95%	*A*_R_	*H*_O_	*H*_*E*_	*F*_IS_	*F*_IS_ low 95%	*F*_IS_ high 95%
GQ	1.534	0.172	0.165	−0.034	−0.056	−0.038	1.291	0.093	0.082	−0.089	−0.117	−0.087
MB	1.539	0.178	0.163	−0.071	−0.124	−0.079	1.328	0.122	0.102	−0.136	−0.212	−0.138
STR	1.525	0.170	0.154	−0.09	−0.200	−0.101	1.342	0.157	0.115	−0.249	−0.479	−0.234
FB	1.546	0.195	0.167	−0.121	−0.198	−0.131	1.365	0.137	0.110	−0.152	−0.255	−0.160
NAM	1.537	0.176	0.157	−0.101	−0.230	−0.111	1.374	0.141	0.107	−0.186	−0.404	−0.193
ANG	1.528	0.171	0.149	−0.137	−0.399	−0.137	1.795	0.476	0.330	−0.413	−0.600	−0.411
Mean	1.535	0.177	0.159	−0.092	−0.201	−0.100	1.416	0.188	0.141	−0.204	−0.345	−0.204

Abbreviations: ANG, Angola; A_*R*_, rarefactioned allelic richness; FB, False Bay; *F*_IS_, inbreeding coefficient, 95% confidence interval limits for *F*_IS_; GQ, Gqeberha; *H*_E_, expected Heterozygosity; *H*_O_, observed Heterozygosity; MB, Mossel Bay; NAM, Namibia; STR, Struisbaai.

Population pairwise *F*_ST_ based on neutral data indicated some shallow but significant differentiation between sampling sites, particularly involving Gqeberha and Struisbaai (Table 2). Overall, differentiation was low with a maximum value of 0.004 between False Bay and Struisbaai. Based on adaptive SNPs, all population pairwise *F*_ST_ values were significant and a magnitude larger than for the whole dataset, ranging from 0.043 to 0.378 between False Bay and Mossel Bay and Angola and Gqeberha respectively (Table 2). Results of the AMOVA showed no evidence for inter‐oceanic structure based on the neutral data with almost all of the genomic variation (99.91%) found within sampling sites and a small, albeit significant part of the total variation among sites within groups (*F*_SC_ = 0.002, *p*‐value = 0.024) (Table 3). Overall, results of the AMOVA based on the adaptive data showed a similar pattern with the majority of genomic variation found within sampling sites (68.8%) but a significant proportion (32.96%) detected among sites within groups (*F*_SC_ = 0.324, *p*‐value = 0.001).

**TABLE 2:** Population pairwise *F*_ST_ in bronze whaler shark *Carcharhinus brachyurus* in Southern Africa.

	GQ	MB	STR	FB	NAM	ANG
GQ	–	**0.074**	**0.154**	**0.095**	**0.114**	**0.378**
MB	**0.001**	–	**0.098**	**0.043**	**0.103**	**0.315**
STR	**0.001**	**0.002**	–	**0.109**	**0.186**	**0.305**
FB	**0.001**	−0.002	**0.004**	–	**0.091**	**0.284**
NAM	0	−0.002	**0.002**	0	–	**0.260**
ANG	−0.002	0.001	**0.003**	**0.002**	0.001	–

*Note*: *F*_ST_ values below diagonal are based on 26,065 neutral SNPs and values above diagonal on 234 adaptive SNPs. 95% confidence intervals (CIs) were generated through 1000 bootstrap intervals. Values in bold indicate significant pairwise variation where the lower bound of the 95% CIs remained above 0.

**TABLE 3:** Analysis of molecular variance (AMOVA) examining the hierarchical partitioning of genetic variation among sampled bronze whaler sharks *Carcharhinus brachyurus* for 26,065 neutral and 234 adaptive SNPs.

Distribution of variation	Neutral data			Adaptive data		
	% var.	Fixation index	*p*‐value	% var.	Fixation index	*p*‐value
Among groups	−0.075	*F*_CT_: −0.001	0.610	−1.787	*F*_CT_: −0.018	0.097
Among sites within groups	0.161	*F*_SC_: 0.002	0.024*	32.958	*F*_SC_: 0.324	0.001***
Within sites	99.914	*F*_ST_: 0.001	0.112	68.829	*F*_ST_: 0.312	0.001***

*Note*: Samples from GQ, MB and STR were assigned to the South Western Indian Ocean group, and samples from FB, NAM and ANG were assigned to the South Eastern Atlantic Ocean group to evaluate genetic differentiation between ocean basins. Significance is indicated as *α = 0.05 and ***α = 0.001.

**FIGURE 3:**
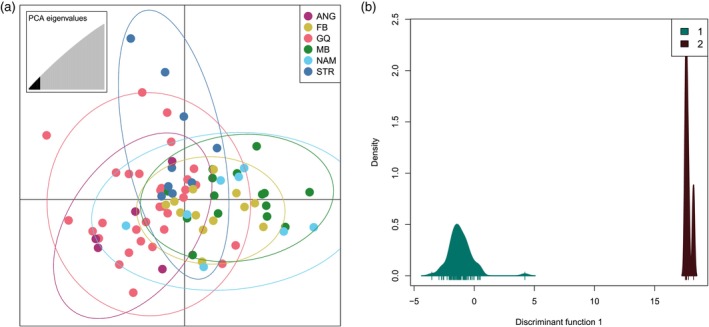
(a) Scatterplot of DAPC with 12 retained principal components as determined through cross‐validation and prior grouping based on sampling site for 26,065 neutral SNPs. (b) DAPC density plot of the distribution of each cluster for *K* = 2 on the discriminant axis with 13 principal components retained based on 234 adaptive SNPs.

**FIGURE 4:**
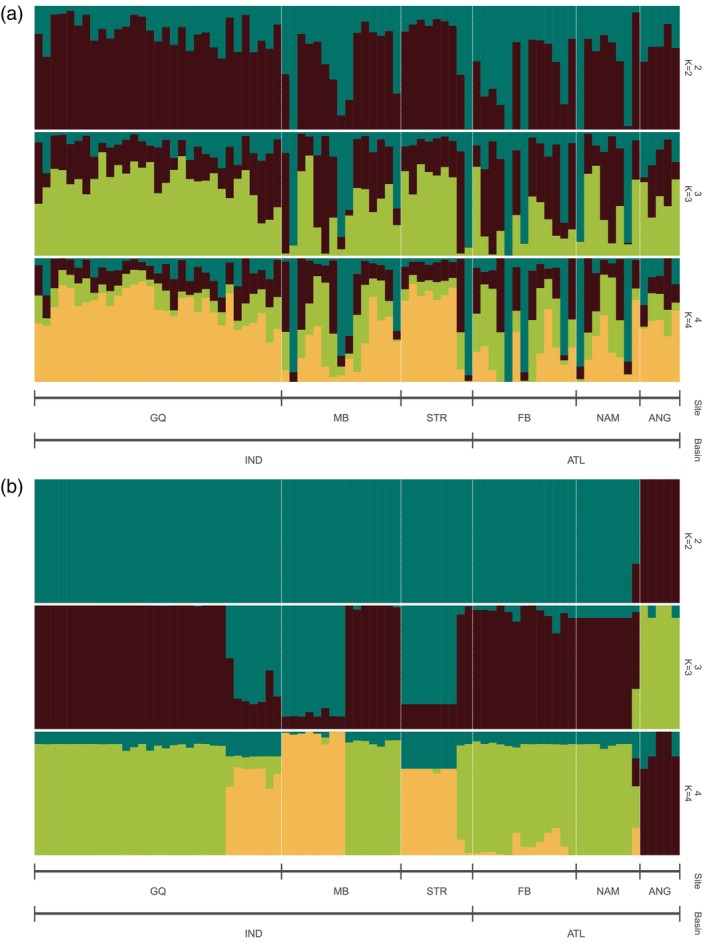
Sparse non‐negative matrix factorization (sNMF) barplots showing individual ancestry coefficients for two to five ancestral populations (*K* = 2–4) based on (a) 26,065 neutral and (b) 234 adaptive SNPs.

When searching for the number of clusters that best describe the neutral dataset using the *k.means* algorithm, the BIC values indicated *K* = 1 (Figure S3a) and mean successful assignment rate using the cross‐validation method, was only 0.26, indicating no population structure (Figure S4a). Similarly, when plotting samples using sampling site as a priori grouping, all samples clustered closely together (Figure 3a). Model selection inferred from the *k*‐means algorithm based on the adaptive data showed a steep decline in BIC value at *K* = 2 with values further decreasing until *K* = 8 (Figure S3b). The cross‐validation showed that retaining 13 PCs achieved the highest predictive success (92%) and lowest root mean squared error (Figure S4b). Evaluating the density plot for *K* = 2 showed a clear separation of the two clusters (Figure 3b) with all samples from Angola assigned to cluster 2 while all other samples were assigned to cluster 1 (Figure S5). Similar results were obtained by the clustering approach sNMF, with the cross‐entropy criterion indicating *K* = 1 as the most likely number of ancestral groups for the neutral data (Figure S6a). Visualization of individual admixture coefficients showed high shared admixture across all samples with no clear assignment to specific clusters regardless of sampling site or ocean basin (Figure 4a). For the adaptive data, cross‐entropy values showed an initial steep decline, indicating *K* = 2 as the optimal number of ancestral groups inferred (Figure S6b). Visualizing individual ancestry coefficients for *K* = 2–4 confirmed this by showing a clear separation of the Angolan genotypes regardless of the number of clusters considered (Figure 4b). With an increasing number of *K*, the assignment to clusters did not follow a geographic pattern with individuals from FB and NAM clustering together with some individuals from GQ and MB.

## 4 DISCUSSION

With the increased availability of genome‐wide SNP data for non‐model species, fine‐scale population genomics assessments have been conducted in a number of shark species, including the application of genome scans to identify patterns of local adaptation (Bernard et al., 2021; Boussarie et al., 2022; Marie et al., 2019; Momigliano et al., 2017; Pazmiño et al., 2018; Portnoy et al., 2015). Here, we provide the first seascape genomics assessment of a shark species along the highly heterogeneous coastline of southern Africa. With the analysis of over 26,000 selectively neutral SNPs, we failed to detect any discernible population structure, confirming our hypothesis of high genetic connectivity along the distribution of *C. brachyurus*. At the same time, two genetic clusters could be detected with the 234 candidate SNPs linked to the regions' environmental heterogeneity. This dataset revealed a deep divergence of individuals sampled in Angola and the rest of the samples from Namibia and South Africa. These results suggest an important role for natural selection in shaping genetic differentiation within this species, and add to accumulating evidence of local adaptation in high‐gene flow species (e.g., Attard et al., 2018; Hoey & Pinsky, 2018; Labrador et al., 2022). These results have important implications for fisheries management and the viability of regional *C. brachyurus* populations under a rapidly changing climate.

### 4.1 Distribution of genomic diversity and population structure based on neutral SNPs

Genetic diversity in terms of heterozygosity was relatively low but evenly distributed with *H*_O_ and *H*_E_ for the neutral data ranging from 0.170 to 0.195 and from 0.149 to 0.167, respectively. Diversity was however notably lower than estimated values for *C. brachyurus* in Australia where observed and expected heterozygosity ranged from 0.23 to 0.29 and 0.21 to 0.37, respectively (Junge et al., 2019). Also in comparison to diversity estimates obtained for other shark species based on SNP data, e.g., the grey reef shark *C. amblyrhynchos* (*H*_O_: 0.193–0.312, *H*_E_: 0.133–0.302; Momigliano et al., 2017), tiger shark *Galeocerdo cuvier* (*H*_O_: 0.25–0.26, *H*_E_: 0.24–0.26; Bernard et al., 2021) and Galapagos shark *C. galapagensis* (*H*_E_: 0.194–0.237; Pazmiño et al., 2018), estimates of *H*_O_ = 0.111 and *H*_E_ = 0.100 for regional *C. brachyurus* were notably lower. We observed no significant differences between *H*_O_ and *H*_E_ while inbreeding levels were negative, indicating significant outbreeding. Although we currently cannot determine whether this is related to historical events or recent demographic changes, the application of more sophisticated demographic modelling approaches could in future shed light on the species' effective population size and demographic history in the region.

Despite the relatively wide geographical scale of sampling in this study, including multiple known biogeographic barriers, there was no strong population structure observed. Population pairwise *F*_ST_ indicated some shallow but significant differentiation between sampling sites, particularly involving Gqeberha and Struisbaai. However, with values ranging from 0.000 to only 0.004, this rather points to the high statistical power of this dataset than to biological meaningful population differentiation. The hypothesis of inter‐oceanic structure was also rejected by the AMOVA with almost all the variation detected within sampling sites. The lack of strong population structure was further confirmed by DAPC and clustering analyses that were not able to assign individuals to respective populations. Model selection based on BIC for the *k*‐means algorithm, as well as cross‐entropy values in the sNMF clearly indicated that the most optimal number of clusters in the data is *K* = 1. Overall, these results confirm that oceanographic barriers including the Angola‐Benguela Front and the Lüderitz Upwelling Cell do not inhibit gene flow in *C. brachyurus*. The Benguela Upwelling zone is known to shape population structure in a number of teleost fish, e.g., *Atractoscion aequidens* (Henriques et al., 2014), bluefish *Pomatomus saltatrix* (Reid et al., 2016) and Leerfish *Lichia amia* (Henriques et al., 2012), leading to deep divergences and even cryptic speciation (Gwilliam et al., 2018). Similarly, smaller coastal elasmobranchs also tend to appear more fragmented and inhibited by oceanographic barriers in the region and elsewhere (Bester‐van der Merwe et al., 2017; Hull et al., 2019; Maduna et al., 2017; Veríssimo et al., 2017). However, body size alone is not always a good predictor for genetic connectivity in elasmobranchs as depth distribution, habitat, environmental tolerance and reproductive behaviour can also play a crucial role (Hirschfeld et al., 2021). While dispersal alone does not equal gene flow, direct evidence of *C. brachyurus* movements across the region has been reported. Using data from a dedicated tag‐recapture program combined with data from the South African cooperative tagging program managed by the Oceanographic Research Institute (ORI), Holtzhausen and Camarada (2007) postulated that adult *C. brachyurus* migrate seasonally between southern Angola and central Namibia for breeding and pupping, suggesting a demographic link between these two populations. The distribution of age‐groups within the tagging data also led the authors to suggest that Baia dos Tigres (just north of the border between Namibia and Angola) is a breeding area for adults, as well as a nursery area that is used by juveniles travelling north from offshore pupping areas off Sandwich Harbor in Namibia. Further, Holtzhausen and Camarada (2007) reported only two *C. brachyurus* crossing the Lüderitz Upwelling Cell out of 10,000 tagged sharks. In a more recent analysis of the ORI data, a total of three sharks were reported crossing the Lüderitz upwelling cell (Rogers et al., 2022), and multiple sharks were recorded travelling between Namibia and southern Angola. In combination with our data, this suggests that while regional movement across oceanographic barriers is present but relatively rare, genetic mixing is high enough to homogenize allele frequencies among populations. Given the limited number of individuals included in our study, this can be confirmed in future work by expanding sample collection. Nevertheless, our results corroborate reports of high genetic connectivity in the few molecular studies that have been conducted on this species to date. Early research based on mitochondrial data revealed at least three distinct groups in the southern hemisphere: Australia‐New Zealand, South Africa‐Namibia and Peru (Benavides et al., 2011). More recently, Junge et al. (2019) confirmed high gene flow across the Australian population using SNP data, although separation of the westernmost Australian individuals from the rest of Australia and New Zealand was detected. These results are also concordant with reports on detailed movement patterns suggesting that *C. brachyurus* form a single biological stock from Western Australia to the coast of New South Wales (Huveneers et al., 2021). Elsewhere, long‐distance migrations have also been observed with the longest recapture distance of at least 2566 km recorded in the Southwest Atlantic (Cuevas et al., 2021), demonstrating that gene flow along vast distances is not restricted by the migration capabilities of the species.

### 4.2 Adaptive divergence in the face of high gene flow

Despite the contrasting assumptions in the underlying models, the different approaches detected a relatively large number of outlier loci in common. In particular, outflank which relies on the concept that loci putatively under selection exhibit higher differentiation between populations than under a neutral distribution is naturally less capable of detecting outliers in systems under high gene flow (Whitlock & Lotterhos, 2015). The comparatively fewer number of outliers detected would therefore be expected. Despite this, 18 and 23 out of 52 candidates detected by outflank were also identified through LFMM and RDA, respectively. By correlating allelic variation with important environmental factors such as sea‐temperature, salinity and chlorophyll concentration, our analyses revealed a deep divergence and possible adaptive potential for this species under a rapidly changing climate.

Different microevolutionary forces (adaptive and non‐adaptive) are at play in shaping the genetic structure of a species, including genetic drift, gene flow, selection and mutation (Allendorf et al., 2010; Tigano & Friesen, 2016). Our results contribute to the growing body of literature documenting patterns of local adaptation despite high levels of gene flow. Gene flow has a multifaceted role in adaptation as it can disrupt the adaptation process if selection is not strong enough to prevent the loss of advantageous alleles and the introduction of maladaptive alleles can reduce population fitness (Alleaume‐Benharira et al., 2006; Bolnick & Nosil, 2007; Lenormand, 2002). On the other hand, gene flow can increase standing genetic variation on which selection can act and increase the speed of adaptation through rapid propagation of beneficial alleles (Barrett & Schluter, 2008; Gosset et al., 2014). The balance of evolutionary drivers including gene flow and migration therefore plays a crucial role, in combination with underlying demographic parameters and the genetic architecture of the relevant traits involved (Barth et al., 2017; Pfeifer et al., 2018; Schaal et al., 2022; Tigano & Friesen, 2016). In marine fishes, it is increasingly documented that large effective population sizes and wide distributions over diverse environments favor the effects of natural selection and minimize the random effect of genetic drift despite the homogenizing effect of gene flow (Cayuela et al., 2020; Conover et al., 2006; Diopere et al., 2018). In particular, when environmental heterogeneity is strong, selective forces can overwhelm the effect of gene flow and in the current study this likely explains the high adaptive divergence of the Angolan population from the rest of the *C. brachyurus* samples. Data on movement and distribution show that *C. brachyurus* typically occurs throughout cool‐ and warm‐temperate to subtropical climates (Drew et al., 2019; Huveneers et al., 2021; Lucifora et al., 2005; Rogers et al., 2022). Southern Angola therefore represents an extreme, but highly variable environment within the species distribution where the south‐flowing Angola Current just north of the ABFZ creates a tropical regime carrying warm and highly saline water (Hardman‐Mountford et al., 2003; Meeuwis & Lutjeharms, 1990). Our results suggest that this environmental heterogeneity, including key factors such as sea surface temperature, salinity and chlorophyll strongly impacts adaptive diversity in *C. brachyurus*. This is perhaps not surprising as temperature has pervasive effects across all levels of biological processes, particularly in ectotherms (Gervais et al., 2018; Santos et al., 2021; Wheeler et al., 2021) and it is the environmental factor most regularly associated with distribution and migration patterns of elasmobranchs (da Silva et al., 2022; Kajiura & Tellman, 2016; Osgood et al., 2021). Similarly, salinity gradients are known to affect habitat suitability and movement in elasmobranchs (Heupel & Simpfendorfer, 2008; Lauria et al., 2015), as well as behaviour and physiology (Dowd et al., 2010; Hammerschlag, 2006; Schlaff et al., 2014). The role of chlorophyll as driver of adaptive divergence is more difficult to ascertain. SNPs significantly associated with chlorophyll were predominantly located on axis 3 of the RDA model, which was not found significant and therefore the role of this variable may be less important. However, primary productivity has been indicated as driver of adaptive divergence in marine invertebrates (Dorant et al., 2020), teleost fish (Cayuela et al., 2020) and dolphins (Amaral et al., 2012; Barceló et al., 2022). While *C. brachyurus* as a top marine predator does not directly feed on phytoplankton, differences in primary productivity will influence abundance and distribution of their prey. It is therefore possible that strong gradients of primary productivity or other collinear variables play a role in these adaptive processes.

To fully understand the adaptive potential of this species, future studies need to confirm whether the candidate SNPs identified here are in fact involved in local adaptation as the correlation alone does not necessarily imply a causal relationship (Whitlock & Lotterhos, 2015). It is currently not known whether outlier SNPs are located in the genes directly affected by selection, or whether they reflect linkage disequilibrium to a neighbouring gene or regulatory region. Ideally, experimental approaches should be conducted to establish a direct link between allelic variation and fitness differences between resident and non‐resident individuals but these are typically only feasible to conduct in some model plant and animal species. Alternatively, a functional approach could be employed to investigate the underlying mechanics of the candidate SNPs identified but this is currently impeded by the paucity of well‐annotated genomes for carcharhinid species. In the future, the more widespread availability of reference genomes will allow for investigations into the underlying functions of the candidate SNPs identified.

### 4.3 Conservation implications

Our study brought insight into neutral and adaptive diversity in a vulnerable elasmobranch that may be pertinent to the adaptive potential of the species under a changing climate and provide useful directions for management strategies. *Carcharhinus brachyurus* is one of the few species commercially exploited in the Southern African region. It is commonly caught in the demersal‐ and linefishery and is one of the top five exploited elasmobranch species in the Southern African demersal shark trade (da Silva et al., 2015; da Silva & Bürgener, 2007). However, in South Africa catches have declined significantly from 100–200 t between 2010 and 2012 to 11–100 t per year in the period from 2013 to 2019 (DFFE, 2022). In Namibia and Angola, *C. brachyurus* is not reported by commercial fisheries and no catch data for this species is available, which may pose a stark threat to the population. Given its imperilled status elsewhere, Southern Africa may host the last stronghold for *C. brachyurus* globally, and as such a precautionary approach to the management and conservation of this species should be taken. Firstly, we can confirm that *C. brachyurus* can be considered a single genetic stock along the Southern African coast based on neutral mutation‐drift processes. This means that oceanographic barriers in the region have little to no effect on gene flow in this species. If population declines were to be observed, the high genetic connectivity uncovered here suggests that management may need to consider fisheries data from across jurisdictional areas. This is particularly relevant considering the recent listing of requiem sharks, which includes *C. brachyurus*, under CITES Appendix S1. This listing imposes regulations on international trade effective from late 2023. Our analyses further demonstrate that the population in Angola carries unique adaptive diversity suggestive of local adaptation to the relatively extreme environmental conditions in the area. Considering the expansion of oxygen‐minimum‐zones (Diaz & Rosenberg, 2008) in combination with warming seas and ocean acidification (Belkin, 2009; Guinotte & Fabry, 2008), differences in adaptive potential may have crucial implications for the conservation of the species under a changing climate. The area between Southern Angola and Northern Namibia has been identified as a particularly fast‐warming hotspot with a rise in ocean temperatures more than three times the global average (Hobday & Pecl, 2014; Lima & Wethey, 2012; Vizy & Cook, 2016). At the same time, the Lüderitz Upwelling Cell is intensifying with sea surface temperatures showing a significant decrease (Narayan et al., 2010). As long‐lived species with slow molecular evolution (Martin et al., 1992; Martin & Palumbi, 1993), elasmobranchs may struggle to evolve fast enough to keep up with the rapid environmental changes. Tracking their environmental niche through migration may be the best option for generalist and wide‐ranging elasmobranchs and some species are already showing distributional shifts in response to changes in environmental conditions (Bangley et al., 2018; Hammerschlag et al., 2022). In light of this, the Angolan population may constitute a crucial pool of adaptive variation that can be spread to the rest of the population. It is therefore advisable to ensure the protection of the Angolan population as it can potentially contribute to the long‐term persistence of the entire stock. The adaptive signal found in the current population also has implications for investigations into future habitat losses and expansions as more realistic projections can be generated when intraspecific adaptive potential is integrated into species distribution models (Bay et al., 2017; Razgour et al., 2019). The results presented here therefore provide a foundation for future investigations to provide a better understanding of the species' vulnerability and potential for adaptation.

## CONFLICT OF INTEREST STATEMENT

The authors declare no conflicts of interest.

## Supporting information


Appendix S1

## Data Availability

The genotype data that support the findings of this study are openly available at figshare (https://doi.org/10.6084/m9.figshare.24516286.v1).
