# Supplementary material for: Local adaptation with gene flow in a highly dispersive shark
Source: Evol Appl. 2023 Dec 20;17(1):e13628. doi: 10.1111/eva.13628 (PMC10810256; doi:10.1111/eva.13628)
Supplement: Supplementary file 1 — Appendix S1 [file EVA-17-e13628-s001.docx]

**Supplementary File:**

**Local adaptation with gene flow in a highly dispersive shark**

Juliana D Klein, Simo N Maduna, Matthew L Dicken, Charlene da Silva, Michelle Soekoe, Meaghen E McCord, Warren M Potts, Snorre B. Hagen, Aletta E Bester-van der Merwe

**3RAD library preparation**

For each sample per 3RAD Design, 20ng of genomic DNA was digested for 1 h at 37 °C in a solution with 1.5 µl of 10x Cutsmart® buﬀer, 0.25 µl (NEB®) of Read 1 enzyme (D1: *XbaI*, D2: *MspI; NEB®*) at 20 U/µl, 0.25 µl of Read 2 enzyme (D1: *EcoRI-HF*, D2: *BamHI-HF*; NEB®) at 20 U/µl, 0.25 µl of Read 1 adapter dimer-cutting enzyme (D1: *NheI*, D2: *ClaI*) at 20 U/ µl, 1 µl of i5Tru adapter at 2.5 µM, 1 µl of i7Tru adapter at 2.5 µM and 0.75 µl of dH_2_O. After digestion/ligation, samples were pooled and cleaned with 1.2x Sera-Mag SpeedBeads (Fisher Scientiifc™) in a 1.2:1 (SpeedBeads:DNA) ratio, and cleaned DNA was eluted in 60 µL of TLE. An enrichment PCR of each sample was carried with 10 µl of 5x Kapa Long Range Buﬀer (Kapa Biosystems, Inc.), 0.25 µl of KAPA LongRange DNA Polymerase at 5 U/µl, 1.5 µl of dNTPs mix (10 mM each dNTP), 3.5 µl of MgCl_2_ at 25 mM, 2.5 µl of iTru5 primer at 5 µM, 2.5 µl of iTru7 primer at 5 µM and 5 µl of pooled DNA. The i5 and i7 adapters ligated to each sample using a unique combination (2 i5 X 1 i7 indexes). The temperature conditions for PCR enrichment were 94 °C for 2 min of initial denaturation, followed by 10 cycles of 94 °C for 20 sec, 57 °C for 15 sec and 72° for 30 sec, and a final cycle of 72 °C for 5 min. The enriched samples were cleaned and quantified with a Quantus™ Fluorometer. Library pools per design were pooled to equimolar concentrations andsent to the Norwegian Sequencing Centre (NSC) for quality control and subsequent final size selection using a one-sided bead clean-up (0.7:1 ratio) to capture 550 bp +/- 10% fragments, and the final paired-end (PE) 150 bp sequencing.

**Supplementary Tables**

**Table S1:** Results of parameter optimization for *denovo* assembly of RAD loci in stacks v2.59 (Catchen et al. 2011). To determine suitable parameter values, a subset of samples (N=14) was used to run the core pipeline several times, iterating over increasing values of the assembly parameters. Optimal parameters (highlighted in yellow) were then identified based on the *r80* rule (Paris et al. 2017; Rochette and Catchen 2017).

| ***Optimization of parameter m with M and n default*** |  |  |  |  |
| --- | --- | --- | --- | --- |
|  |  |  |  |  |
| value of m | assembled loci | **r80** polymorphic loci | number of snps | NEW polymorphic loci |
| 3 | 207694 | 66737 | 85173 | 66737 |
| 4 | 188220 | 59131 | 76451 | -7606 |
| 5 | 171482 | 52650 | 68544 | -6481 |
| 6 | 156328 | 46821 | 61000 | -5829 |
| 7 | 141863 | 41183 | 53588 | -5638 |
|  |  |  |  |  |
|  |  |  |  |  |
| ***Optimization of parameter M with n=M and m=3*** |  |  |  |  |
|  |  |  |  |  |
| value of M | assembled loci | **r80** polymorphic loci | number of snps | NEW polymorphic loci |
| 1 | 213001 | 67135 | 82596 | 67135 |
| 2 | 208396 | 67552 | 85775 | 417 |
| 3 | 204921 | 67157 | 86310 | -395 |
| 4 | 201665 | 66262 | 86752 | -895 |
| 5 | 198814 | 65402 | 87024 | -860 |
| 6 | 196300 | 64681 | 86998 | -721 |
| 7 | 194033 | 64076 | 87957 | -605 |
| 8 | 192100 | 63557 | 88390 | -519 |
| 9 | 190247 | 63102 | 90044 | -455 |
|  |  |  |  |  |
|  |  |  |  |  |
| ***Optimization of parameter n with m=3 and M=2*** |  |  |  |  |
|  |  |  |  |  |
| value of n | assembled loci | **r80** polymorphic loci | number of snps | NEW polymorphic loci |
| 1 | 207688 | 66743 | 85190 | 66743 |
| 2 | 232020 | 77561 | 96438 | 10818 |
| 3 | 208365 | 68392 | 87620 | -9169 |

**Table S2:** Environmental parameters for six sites across Southern Africa where bronze whaler sharks were sampled. Sea-surface temperature (sst_min, sst_max, sst_mean, sst_range between 2002 and 2010) and salinity data (sss_min_sss_max, sss_mean, sss_range between 1955 and 2006) for the period were obtained from the marspec database at a resolution of ~ 1 km. Mean surface dissolved oxygen, pH and chlorophyll concentration for the period of 2000 to 2014 were retrieved from bio-oracle at a resolution of ~9.2 km.

| Site | lat | long | sst_min  (C°) | sst_max  (C°) | sst_mean  (C°) | sst_range  (C°) | sss_min  (PSU) | sss_max  (PSU) | sss_mean  (PSU) | sss_range  (PSU) | Oxygen  (mmol m 3) | pH | Chlorophyll  (mg m 3) |
| --- | --- | --- | --- | --- | --- | --- | --- | --- | --- | --- | --- | --- | --- |
| GQ | 34.061 | 25.210 | 16.755 | 21.400 | 18.749 | 4.645 | 35.173 | 35.480 | 35.310 | 0.308 | 230.760 | 8.229 | 1.133 |
| MB | 34.300 | 22.025 | 15.221 | 21.873 | 18.264 | 6.652 | 35.260 | 35.480 | 35.362 | 0.220 | 238.197 | 8.227 | 0.813 |
| STR | 34.768 | 20.187 | 14.822 | 20.598 | 17.212 | 5.783 | 35.230 | 35.412 | 35.322 | 0.182 | 238.569 | 8.227 | 0.771 |
| FB | 34.161 | 18.710 | 14.202 | 19.019 | 16.519 | 4.815 | 35.251 | 35.441 | 35.332 | 0.190 | 242.892 | 8.227 | 1.348 |
| NAM | 22.065 | 14.116 | 13.360 | 20.190 | 16.170 | 6.830 | 34.920 | 35.300 | 35.110 | 0.380 | 225.491 | 8.196 | 3.125 |
| ANG | 16.259 | 11.720 | 15.890 | 22.180 | 19.030 | 6.290 | 35.500 | 35.860 | 35.680 | 0.360 | 212.699 | 8.201 | 1.615 |

Supplementary Figures


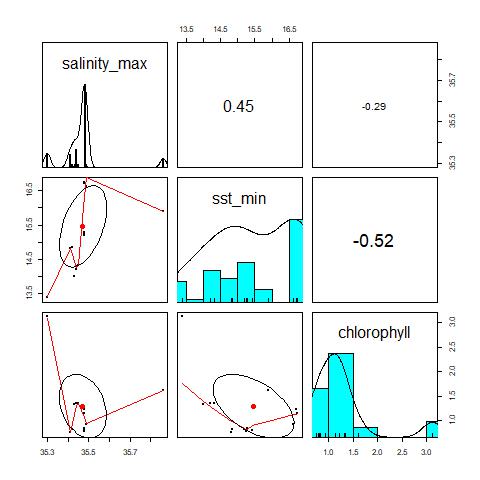


**Figure S1:** Correlation coefficients between selected environmental variables. No variables had correlation coefficient values of r > 0.7, hence all three were retained for further analysis.

**
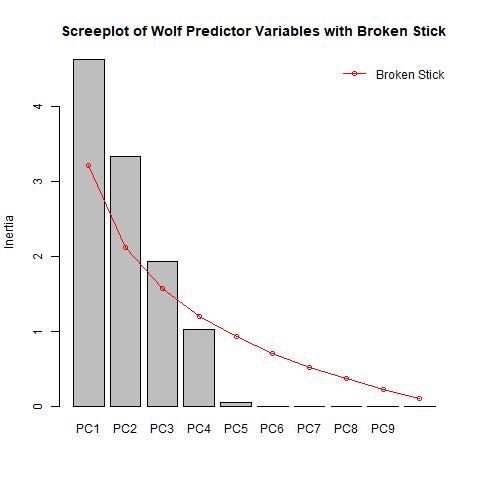
**

**Figure S2:** Screeplot showing eigenvalues of principal components of 11 environmental variables used in a latent factor mixed model. The red line indicates the expected values generated by a random distribution (“Broken-Stick”).

**
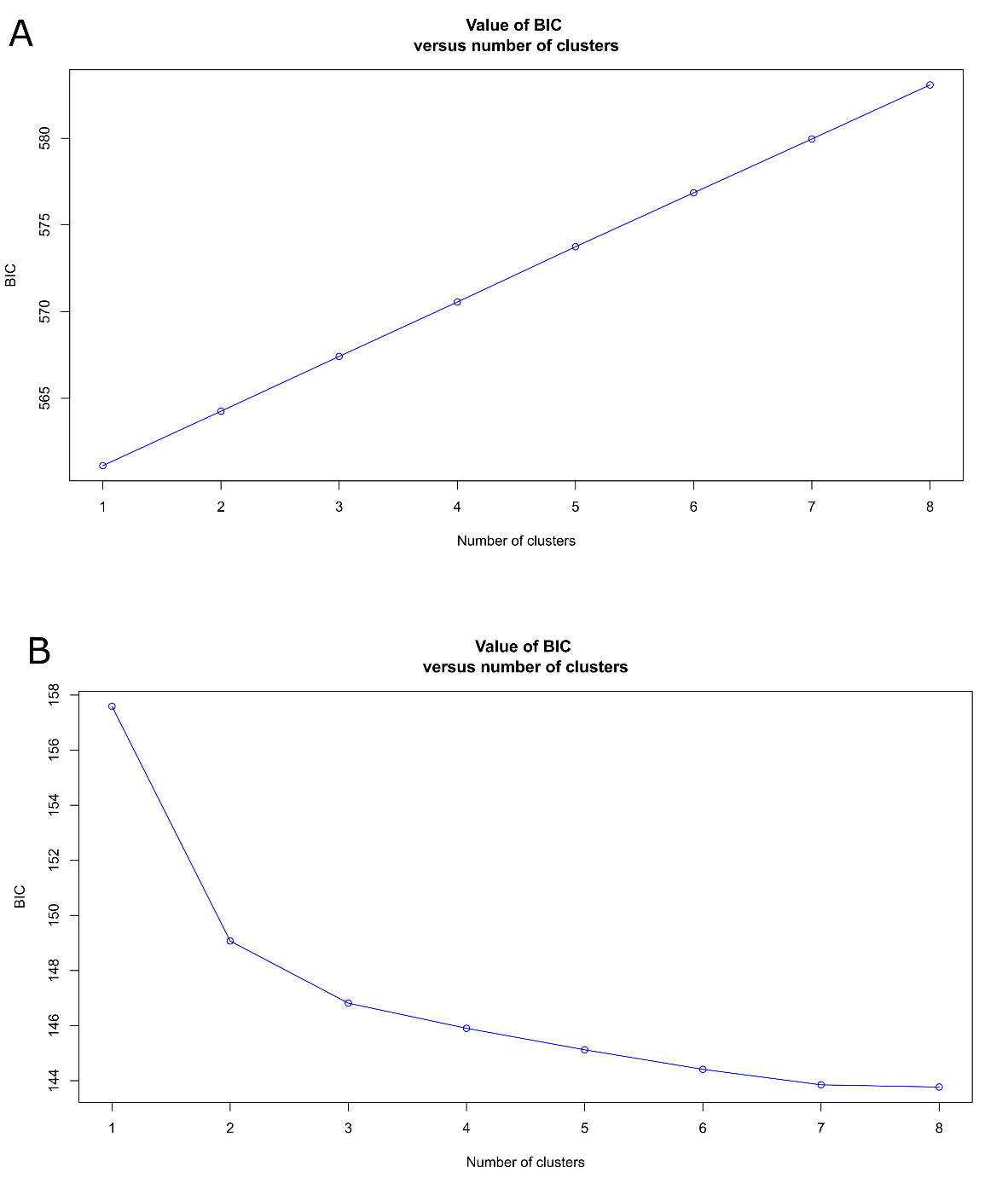
**

**Figure S3:** Model selection based on the Bayesian Information Criterion (BIC) for increasing number of clusters as inferred by the k-means algorithm implemented in the function find.clusters. Analysis in (A) was based on 26,065 neutral SNPs and (B) was based on 234 adaptive SNPs.

**
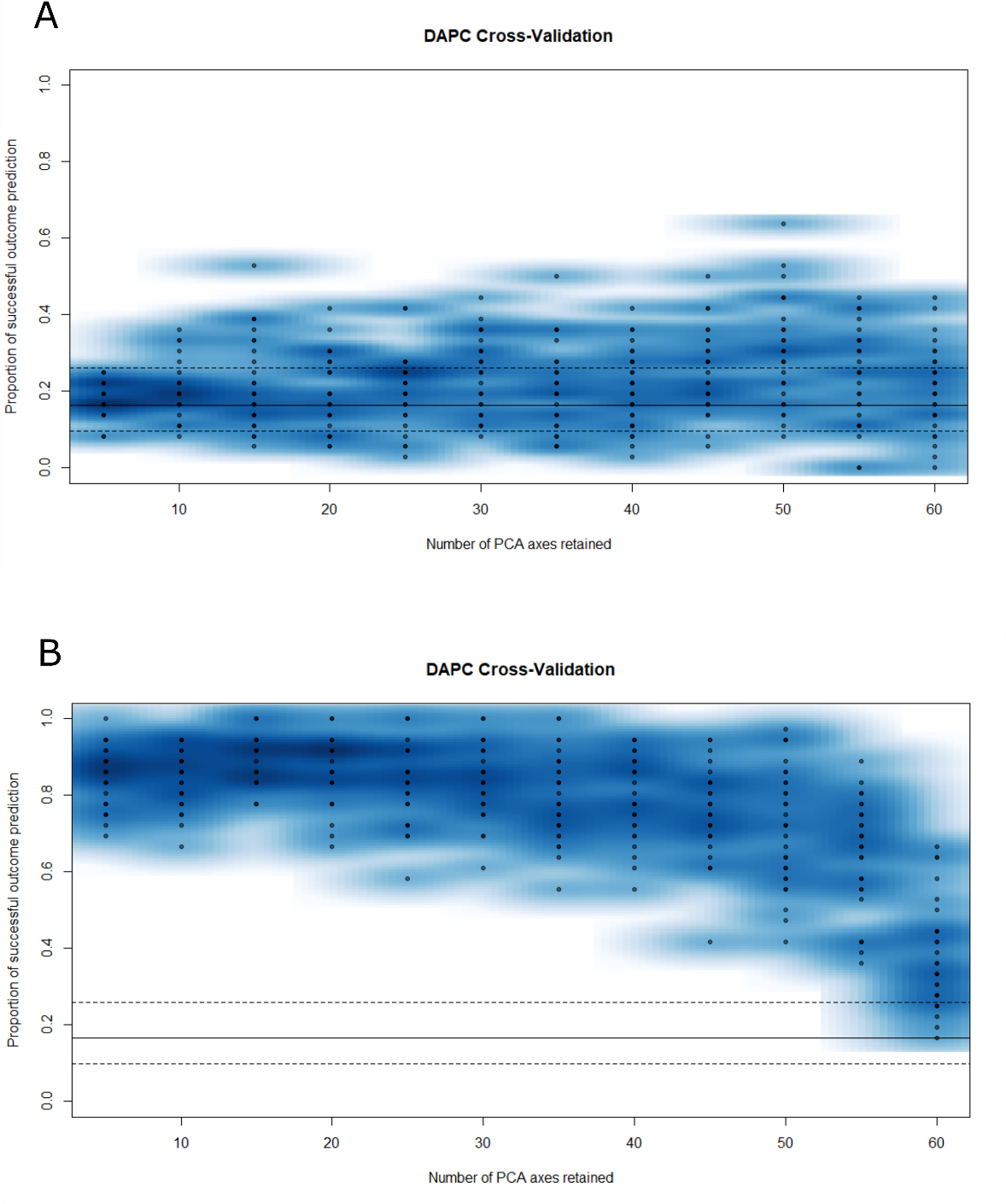
**

**Figure S4:** Proportion of successful outcome predictions from cross-validation procedure using a split dataset (90% training, 10% validation data) where DAPC was repeated 1,00 times with varying number of PCs retained. The highest predictive success associated with lowest root squared mean error was obtained by retaining 12 PCs for the neutral dataset (26,065) (A) and 13 PCs for the adaptive dataset (234 SNPs) (B).


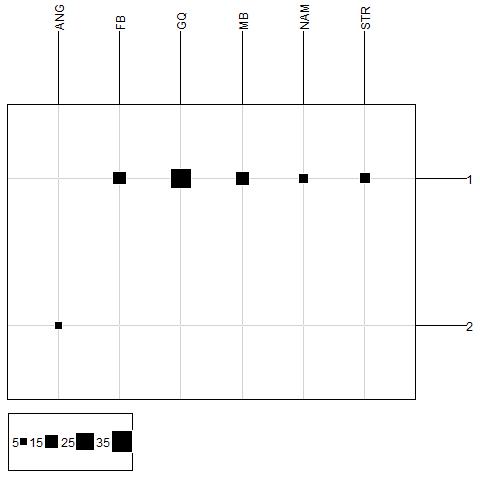


**Figure S5:** DAPC results for 234 adaptive SNPs showing assignment table with columns corresponding to sampling sites and rows corresponding to inferred clusters for K = 2. All individuals from Angola are assigned to cluster 2, while cluster 1 contains the rest of the samples.

**
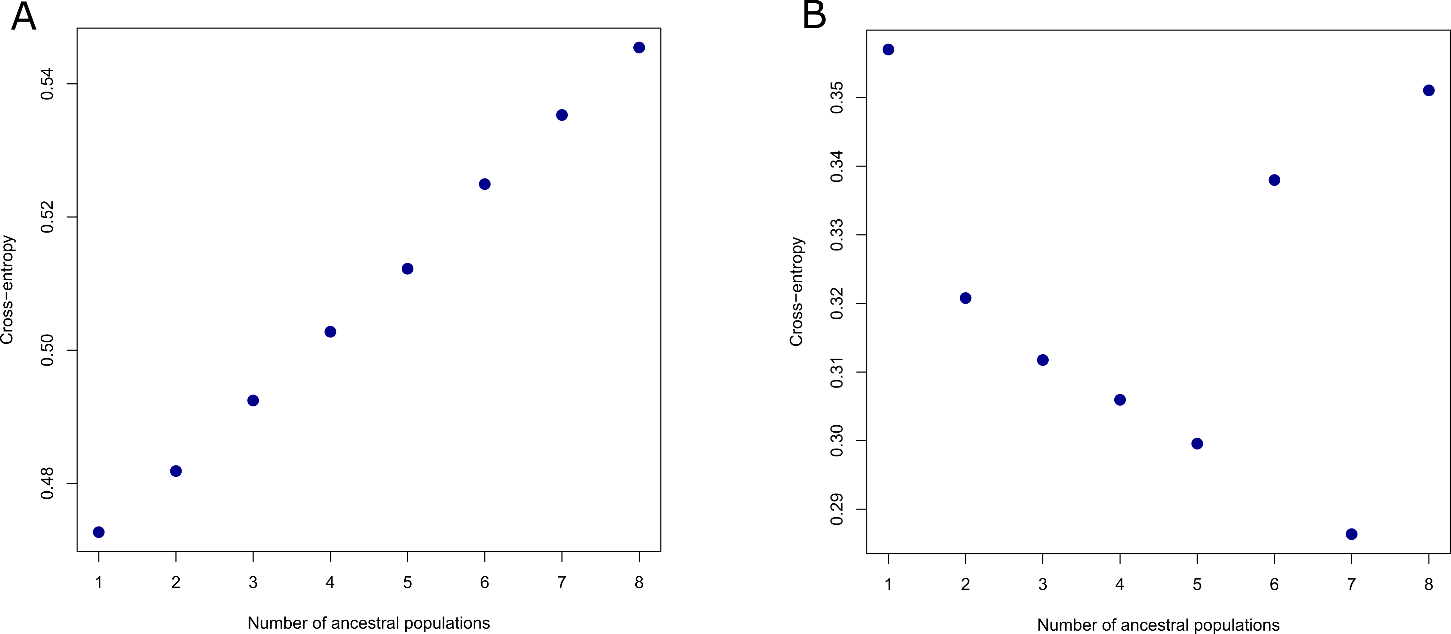
**

**Figure S6:** Cross-entropy values for varying number of ancestral populations (K=1-8) estimated in spare non-negative matrix factorization (sNMF) analysis. The replicate with the lowest cross-entropy per K-value is shown here for the neutral dataset (26,065 (SNPs) (A) and 13 PCs for the adaptive dataset (234 SNPs) (B).
